# Human NK Cells in Autologous Hematopoietic Stem Cell Transplantation for Cancer Treatment

**DOI:** 10.3390/cancers13071589

**Published:** 2021-03-30

**Authors:** Ane Orrantia, Iñigo Terrén, Gabirel Astarloa-Pando, Olatz Zenarruzabeitia, Francisco Borrego

**Affiliations:** 1 Immunopathology Group, Biocruces Bizkaia Health Research Institute, 48903 Barakaldo, Spain; ane.orrantiarobles@osakidetza.eus (A.O.); inigo.terrenmartinez@osakidetza.eus (I.T.); gabirel.astarloapando@osakidetza.eus (G.A.-P.); 2 Ikerbasque, Basque Foundation for Science, 48013 Bilbao, Spain

**Keywords:** NK cells, hematopoietic stem cell transplantation (HSCT), autologous HSCT, graft versus tumor, graft versus leukemia, KIR, HLA, multiple myeloma, non-Hodgkin lymphoma

## Abstract

**Simple Summary:**

Natural killer (NK) cells are key elements of the innate immune system that have the ability to kill transformed (tumor and virus-infected) cells without prior sensitization. Hematopoietic stem cell transplantation (HSCT) is a medical procedure used in the treatment of a variety of cancers. The early reconstitution of NK cells after HSCT and their functions support the therapeutic potential of these cells in allogenic HSCT. However, the role of NK cells in autologous HSCT is less clear. In this review, we have summarized general aspects of NK cell biology. In addition, we have also reviewed factors that affect autologous HSCT outcome, with particular attention to the role played by NK cells.

**Abstract:**

Natural killer (NK) cells are phenotypically and functionally diverse lymphocytes with the ability to recognize and kill malignant cells without prior sensitization, and therefore, they have a relevant role in tumor immunosurveillance. NK cells constitute the main lymphocyte subset in peripheral blood in the first week after hematopoietic stem cell transplantation (HSCT). Although the role that NK cells play in allogenic HSCT settings has been documented for years, their significance and beneficial effects associated with the outcome after autologous HSCT are less recognized. In this review, we have summarized fundamental aspects of NK cell biology, such as, NK cell subset diversity, their effector functions, and differentiation. Moreover, we have reviewed the factors that affect autologous HSCT outcome, with particular attention to the role played by NK cells and their receptor repertoire in this regard.

## 1. Introduction

Natural killer (NK) cells constitute an essential part of the innate immune system and probably are the best-known members of the heterogeneous family of the innate lymphoid cells (ILCs). In contrast to T and B lymphocytes, ILCs lack rearranged antigen-specific receptors and act early in the immune response [1]. ILCs are classified into five different subsets based on their transcriptional profile and functions: ILC1, ILC2, ILC3, lymphoid tissue-inducer (LTi) cells, and NK cells [2]. Moreover, ILCs can be grouped into cytotoxic ILCs and non-cytotoxic “helper” ILCs. NK cells represent the classic cytotoxic population of ILCs [3,4]. As ILC1s, NK cells also produce the pro-inflammatory cytokine interferon (IFN)-γ but are dependent on both T-bet and eomesodermin (Eomes) transcription factors to develop, while ILC1s only depend on T-bet [5,6]. Even if generally classified into the abovementioned five subsets, several studies have also demonstrated that ILCs can transdifferentiate into other ILC subsets depending on the cytokine milieu, evidencing the plasticity and the heterogeneity of these cells [7,8,9,10].

Upon activation, NK cells can mediate different effector mechanisms to eliminate both normal and transformed (tumor and virus-infected) cells [11]. NK cell activation may trigger the release of cytolytic granules to the immunological synapse leading to the killing of target cells. These granules are composed of perforin and granzymes. Perforin is a pore-forming molecule that facilitates granzymes to enter into the target cells [12]. Then granzymes initiate target cell apoptosis by different pathways. For instance, granzyme B can cleave pro-caspases into active caspases inducing caspase-dependent cell death or can activate mitochondrial death pathway [11,13]. In addition to the perforin/granzyme pathway, NK cells also express FasL and TRAIL, which are ligands for tumor-cells expressing Fas (CD95) and TRAIL receptors, members of the tumor necrosis factor (TNF) receptor superfamily. The engagement of these death receptors with their corresponding ligands also contributes to NK cell cytotoxicity by activating the caspase enzymatic cascade that causes the apoptosis of the target cell [11,14,15]. Moreover, the anti-tumor and anti-viral activity of NK cells also involves the secretion of cytokines, as, e.g., IFN-γ, which act on tumor and virus-infected cells [16]. Finally, NK cells through their cytotoxic activity and secretion of cytokines and chemokines also have a very important role in shaping the innate and adaptive immune responses [17].

Many studies performed in mice support the notion that NK cells play a critical role in the eradication of tumor cells [18,19,20]. In humans, in an 11-year follow-up study, it was found that low natural cytotoxic activity of peripheral-blood mononuclear cells was associated with increased cancer risk [21]. Moreover, in acute myeloid leukemia (AML) patients receiving alloreactive NK cells in the course of allogenic hematopoietic stem cell transplantation (alloHSCT), a reduced incidence of relapse was observed in patients who lacked the human leukocyte antigen (HLA) ligands for donor inhibitory killer cell immunoglobulin-like receptors (KIRs) [22]. Thus, these data indicate a significant role for NK cells in tumor surveillance.

## 2. NK Cell Subset Diversity

Traditionally, according to the differential expression of CD56 and CD16, human NK cells are divided in two major subsets known as CD56^bright^CD16^low/−^ and CD56^dim^CD16^+^ (hereafter referred to as CD56^bright^ and CD56^dim^, respectively) [23]. Although both subsets express similar levels of several surface receptors such as the common interleukin (IL)-2 and IL-15 receptor beta chain (IL-2/15Rβ), NKG2D, and NKp80, there are some distinct phenotypical and functional features between them [24]. For instance, CD56^bright^ NK cells express CD94/NKG2A (hereafter NKG2A) but lack the expression of KIRs. They also show low expression of perforin and granzymes A and B and low or undetectable expression of CD16. In addition, they produce large amounts of immunomodulatory cytokines and chemokines in response to cytokines but are poor mediators of direct cytotoxicity and antibody-dependent cell-mediated cytotoxicity (ADCC) [23,25,26]. However, it has been also demonstrated that after cytokine activation they become more cytotoxic, as, e.g., upon priming with IL-15 and after expansion with K562-mb-IL21 feeder cells, the CD56^superbright^CD16^+^ NK cells exhibit increased degranulation after target recognition [27,28]. Conversely, CD56^dim^ NK cells are characterized by lower expression of NKG2A and high expression of CD16, perforin, granzymes, and KIRs. Moreover, CD56^dim^ NK cells are highly cytotoxic but produce lower amounts of cytokines in response to monocyte-derived cytokines [23,25]. Nevertheless, they can produce significant amounts of cytokines upon target cell recognition [29,30]. CD56^dim^ NK cells that lack CD16 expression have also been identified in patients undergoing haploidentical HSCT. Due to their transcriptional profile, these cells, also known as unconventional CD56^dim^ NK cells, are considered to be an intermediate stage between CD56^bright^ and CD56^dim^ NK cells [31]. Finally, CD56^bright^ and CD56^dim^ NK cells also exhibit different expression patterns of chemokine and homing receptors, which are linked to their tissue localization [32]. CD56^bright^ are the predominant NK cell subsets in secondary lymphoid tissues (SLT), while CD56^dim^ are the main subset in the peripheral blood [23,25].

However, results published over the last years have revealed that human NK cells are much more diverse than just CD56^bright^ and CD56^dim^ subsets. A study using mass cytometry that simultaneously analyzed more than 30 parameters revealed that, within an individual, there may be 6000–30,000 phenotypically distinct peripheral blood NK cells, highlighting the existing NK cell diversity [33]. Furthermore, recently, the diversity of peripheral blood NK cells has also been revealed by single-cell RNA sequencing analysis [34]. This phenotypic diversity is mainly influenced by genetics and environmental factors. Genetics strongly influence the combinatorial expression patterns of KIRs that recognized HLA class I molecules. Moreover, different environmental factors influence the expression of activating and costimulatory receptors [33,35]. For example, HLA-DR expression varies in response to activation [36,37], the expression of CD117 differs during differentiation [38] and the expression of 2B4 could be regulated in response to viral infection [39]. Furthermore, different tissue-resident NK cell subpopulations have been identified in different peripheral tissues, such as human liver, uterus, spleen, intestine, and salivary gland, showing that local tissue environment also contributes to NK cell diversity [35,40]. These tissue-resident NK cells are in general CD56^bright^, express tissue-residency markers (e.g., CD49a, CD69, and CD103), which are involved in retaining lymphocytes in tissues, and present unique transcriptional signatures distinct from peripheral blood NK cells [25,41].

Infections by different pathogens also contribute to NK cell diversity. For instance, infection with human cytomegalovirus (CMV), a DNA virus that belongs to the herpesvirus family, is associated with important changes in the configuration of the NK cell repertoire [42]. Increased numbers of CD94/NKG2C+ (hereafter NKG2C+) NK cells, commonly known as adaptive NK cells, have been observed following CMV infection or reactivation in different clinical settings [43,44,45]. These adaptive NK cells exhibit memory properties such as increased CD16-triggered responses [46]. CMV infection is also associated with an increase in CD57 expression on NK cells [47]. Although NKG2C+ NK cells expansion has been observed in patients infected by other viruses, it only occurs in individuals who have been previously infected with CMV, thus suggesting that the expansion of these cells might be specific to human CMV [42,46]. On the other hand, adaptive NK cells subsets are also characterized by the lack of expression of FcεRγ, and they also exhibit an enhanced ADCC. These FcεRγ-negative adaptive NK cells have been described to be expanded in CMV infection, although they can be found in CMV seronegative individuals as well [46,48]. Furthermore, even though the lack of FcεRγ is often associated with NKG2C expression, both markers appear occasionally disassociated, suggesting the existence of distinct human adaptive NK cell subsets [49].

Additionally, other viral infections (e.g., human immunodeficiency virus (HIV)-1) and also aging, alter NK cell subsets repertoire. An expansion of CD56^neg^CD16^+^ NK cell subset (hereafter CD56^neg^) has been described in both situations [47,50,51]. CD56^neg^ NK cells are very scarce in the peripheral blood of healthy individuals and their expansion occurs primarily at the expenses of CD56^dim^ NK cells [52]. Proteome analysis of CD56^neg^ NK cells from healthy individuals revealed that this subset has an overall CD56^dim^-like phenotype [53]. Moreover, they have been classically described as dysfunctional, even though some studies suggest that the effector functions of CD56^neg^ NK cells, although lower compared to CD56^dim^ NK cells, are not as diminished as previously thought [54,55,56]. The differences among studies may be due to an inadequate identification of this cell subset [55,57]. More age-related alterations have also been described such as a reduced frequency of CD56^bright^ NK cells in elderly individuals and decreased expression of receptors such as NKp30, NKp46, and DNAM-1 on CD56^dim^ NK cells. Furthermore, aging affects NK cell cytotoxicity against classic NK cell targets while ADCC is maintained [58]. Lastly, compared to children, adults show lower developmental marker diversity on NK cells [59].

The necessity to clarify and better understand the NK cell heterogeneity is very evident when novel NK cell subsets that have an impact on the prognosis of different pathological settings are identified. For instance, Stannard et al. identified a new terminally differentiated CD56^dim^ NK cell subset that lack expression of DNAM-1 and, among others, present low production of IFNγ and limited killing capacities. These authors reported a decline in the ratio of CD56^dim^DNAM-1^+^/CD56^dim^DNAM-1^neg^ NK cell subsets and a reduced cytotoxicity of CD56^dim^DNAM-1^+^ NK cells in the blood of patients with Hodgkin lymphoma and diffuse large-B cell lymphoma that may have clinical implications [60]. Moreover, recently, a new subset of cord blood-derived NK cells with an immature phenotype, poor effector functions and low diversity index was associated with relapse after cord blood transplantation [61].

## 3. Human NK Cell Receptors and Effector Functions

**Figure 1:**
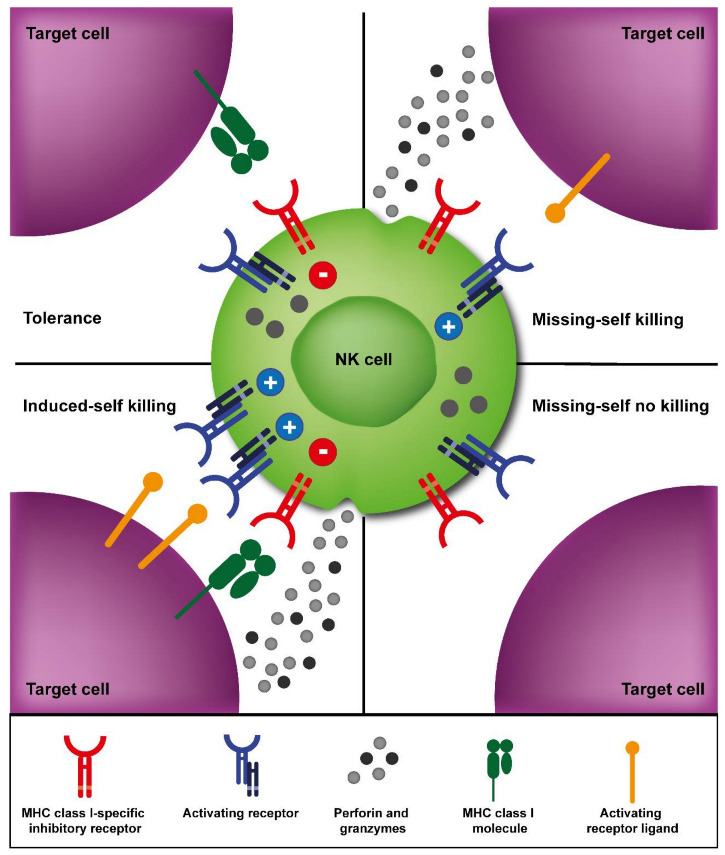
Natural killer (NK) cell activation is determined by an intricate balance between activating and inhibitory signals transmitted by surface receptors. Target cells expressing major histocompatibility complex (MHC) class I molecules that engage MHC class I-specific inhibitory receptors expressed by NK cells will not be killed (tolerance) due to inhibitory signals transmitted by the inhibitory receptors. Target cells that display an upregulation of activating receptor ligands will be killed (induced-self killing) as activating signals will overcome the inhibitory signals. Target cells that downregulate MHC class I molecules will be eliminated depending on the presence of signals transmitted by the activating receptors (missing-self). After activation, NK cells will mediate different effector mechanisms to eliminate target cells, such as the release of cytolytic granules containing perforin and granzymes.

The capacity to recognize and spontaneously eliminate virus-infected and tumor cells is one of the key functions of NK cells. This function is tightly regulated by a wide range of receptors expressed at the cell surface of NK cells [62]. One type of receptors expressed on the surface of NK cells are inhibitory receptors, which can be divided in two main groups. The first group comprise KIRs, which signal upon binding to classical major histocompatibility complex (MHC) class I molecules HLA-A, -B, and -C (Table 1) [63]. The second group comprise the C-type lectin-like heterodimeric receptor NKG2A (and its isoform NKG2B) which recognize the non-classical MHC class I molecule HLA-E [64]. Via inhibitory receptors, NK cells are able to detect the absence of self-molecules, specifically destroying cells that have downregulated HLA class I molecules, a frequent event in cancer cells (Figure 1). This is known as the missing-self recognition [65]. Moreover, NK cells may exert anti-tumor effect due to KIR-HLA class I mismatch. As demonstrated in AML patients undergoing HLA-mismatched alloHSCT, HLA class I-binding receptors of donor NK cells do not engage with their cognate ligand on recipient cells and thus, sense the missing expression of self-HLA class I molecules [66,67,68]. In this scenario, the inhibitory signal mediated by inhibitory receptors upon engagement with their ligands will be absent, thus allowing the killing of the target cells by donor NK cells. Also, and to avoid NK cell activation against self-healthy cells, during development, NK cells must randomly express at least one inhibitory receptor matching the own MHC class I molecules. Otherwise, NK cells become somehow anergic in a process known as NK cell “education” or “licensing” [69]. NK cells can also express other additional constitutive or inducible inhibitory receptors that serve as immune checkpoints for cell activation, (e.g., PD-1, TIGIT, CD96, CD300a, etc.). Upon interaction with their ligands, these receptors hamper NK cell IFN-γ production and NK cell-mediated cytotoxicity [70,71].

However, there are some situations that cannot be explained by the abovementioned missing-self hypothesis. For example, self-cells lacking MHC class I expression, such as human erythrocytes, are not eliminated by NK cells. Moreover, under some circumstances, NK cells are able to eliminate MHC class I-expressing tumor cells. This is because NK cells also have the capability to detect stress-induced ligands present on transformed cells, leading to the postulation of the “induced-self” model [72]. The recognition of those inducible ligands is mediated by a broad spectrum of activating receptors present on the surface of NK cells. Natural cytotoxicity receptors (NCR) are one of the family of activating receptors. These NCRs include NKp46, NKp30, and NKp44, being the last one only expressed after activation of NK cells, while NKp46 and NKp30 are constitutively expressed [73]. Ligands for this family of receptors have been detected not only on many tumor cells but also in some healthy tissues [74]. Other well-studied and particularly important activating receptor is the C-type lectin-like activating receptor NKG2D, which is expressed as a homodimer in virtually all NK cells. In humans, two families of NKG2D ligands (NKG2DLs) have been described: the MHC class I chain-related molecules A and B (MICA and MICB) and the UL16-binding proteins (ULBP). NKG2DLs are, in general, not considered to be expressed on healthy tissues, instead their expression is induced by cellular stress [75]. Other activating receptors expressed by NK cells are the heterodimeric receptor CD94/NKG2C, that interacts with HLA-E and DNAM-1, which compete with TIGIT and CD96 for binding to its ligands PVR (CD155) and Nectin-2 (CD112). Moreover, the function of these activating receptors can be enhanced by a second family of activating co-receptors (e.g., 2B4 and NKp80) whose action requires the co-engagement of the main activating receptors [70].

Thus, the intricate balance between activating and inhibitory signals transmitted by the surface receptors determines if NK cells will be activated and, consequently, what will be the outcome of the encounter with the potential target cell. Healthy cells expressing self-MHC class I molecules and none or few activating ligands will not be attacked by NK cells (tolerance), while transformed cells that present a reduced expression or mismatch expression of MHC class I molecules (missing-self) and/or an increased expression of activating ligands (induced-self) will be eliminated (Figure 1) [76]. Moreover, NK cells are also strongly activated via CD16 surface receptor that recognize the Fc portion of IgG antibodies, and thus are able to kill antibody-coated cells via ADCC. Apart from activating and inhibitory receptors, NK cell activation is also regulated by cytokines secreted by other cells, via the engagement of cytokine receptors present on the surface of NK cells. Particularly, IL-15 is a critical cytokine for NK cell differentiation [77]. Moreover, IL-2 and IL-15 are needed for NK cell survival and proliferation [78]. Furthermore, cooperative effect of IL-2 and IL-15 and other cytokines such as IL-12 and IL-18 induce high IFNγ production by NK cells [79].

## 4. NK Development and Differentiation

The development of NK cells, as it occurs with other leukocytes, comprise a succession of coordinated differentiation steps that lead to commitment toward NK cell lineage and to the acquisition of functional competency. Traditionally, bone marrow has been considered the major site of NK cell generation and differentiation. However, studies carried out over the last years describing the presence of different NK cell progenitors in tissues other than bone marrow evidence that NK cell development also occurs in extra-medullary sites [80]. Thus, it is now well accepted that NK cells can also migrate, develop, and mature in SLT, as is the case of lymphoid nodes [81,82].

Due to obvious experimental limitations, a detailed picture of human NK cell development has been difficult to obtain. Moreover, the classical developmental model in where hematopoietic stem cells (HSCs) bifurcate into common myeloid progenitors (CMP) and common lymphoid progenitor (CLP) was challenged by the discovery of a lymphoid-primed multipotent progenitor (LMPPs) that sustains lymphoid and myeloid potential [83]. The definition of a lineage-restricted NK cell progenitor has been also difficult to achieve given the complexity and plasticity of early hematopoiesis and lineage commitment. Nevertheless, phenotypically distinct progenitors that give rise to NK cells have been described [84,85,86,87,88,89,90]. Years ago, a step forward in the characterization of the human NK cell developmental pathway was done by Caligiuri and colleagues, by defining a five-stage model of human NK cell development in SLT [91,92]. In this study, and based on the differential expression of CD34, CD117, CD94, and CD16, five human NK cell developmental stages were defined as follows: stage 1 CD34^+^CD117^−^CD94^−^CD16^−^, stage 2 CD34^+^CD117^+^CD94^−^CD16^−^, stage 3 CD34^−^CD117^+^CD94^−^CD16^−^, stage 4 CD34^−^CD117^+/−^CD94^+^CD16^−^, and stage 5 CD34^−^CD117^−^CD94^+/−^CD16^+^ [93]. In this model, cells comprising stages 1 and 2 are capable to differentiate into dendritic cells (DCs), T cells, and NK cells, while stage 3 cells can give rise to NK cells but not to DCs or T cells. Thus, stage 3 cells are considered lineage-restricted NK cell precursors. The acquisition of CD122 (IL-2/15 receptor β-chain) marks an important step towards NK cell differentiation as the *trans*-presentation of soluble IL-15 is essential for mature NK cell survival [77,78,94]. However, functional maturity is not acquired until stage 4, as stage 3 cells are characterized by the lack of key mature NK cell features, such as, IFNγ production and perforin-mediated cytotoxicity [93,95].

Subsequent studies have described additional differentiation steps of this model. For instance, using 10-parameters flow cytometry, Eissen et al. defined 7 human NK cell developmental stages in the bone marrow, adding a layer of complexity. They propose that stage 3 can be subdivided in two additional stages distinguished by NK cell lineage commitment acquisition through CD56 expression, stage 3a (CD34^−^CD117^+^CD94^−^CD56^−^) and stage 3b (CD34^−^CD117^+^CD94^−^CD56^+^). Moreover, they also divided stage 5 in two more stages, stage 5a (CD34^−^CD117^−^CD56^+^CD94^+^) and stage 5b (CD34^−^CD117^−^CD56^+^CD94^−^) differentiated by the loss of CD94 expression [80]. In addition, Scoville et al. proposed that SLT stage 2 cells can be divided in two functionally distinct subsets according to surface expression of the IL-1β receptor (IL-1R1) and suggest that stage 2b IL-1R1+ cells represent common ILC progenitors (CILP) in humans [96,97]. Furthermore, two different steps of stage 4 have been described also in SLT, differing in the C-type lectin-like activating receptor NKp80 expression. Stage 4a subset was NKp80- and characterized by ILC3-associated features and not significant effector functions, while stage 4b subset was NKp80+ and cells produced IFNγ and mediated perforin-dependent cytotoxicity [95]. These stage 4b cells are thought to represent CD56^bright^ NK cells, whereas stage 5 cells represent the CD56^dim^ NK cell subset. Thus, according to this linear model of NK cell development, there is a transition from CD56^bright^ to CD56^dim^ defined by, among others, downregulation of CD56 and acquisition of CD16 [84,98]. However, this step is probably controversial, as the existence of CD56^dim^ NK cells was reported in primary immunodeficiency patients that lack CD56^bright^ NK cells [99]. Nevertheless, the generation of CD56^dim^ NK cells from CD56^bright^ NK cells when human HSCs are engrafted in humanized mice [77], the fact that CD56^bright^ NK cells display longer telomeres [100] and that CD56^bright^ NK cells are the predominant population after HSCT [101], among others, supports the hypothesis for a developmental relationship between CD56^bright^ and CD56^dim^ NK cells.

CD56^dim^ NK cells are a very heterogeneous population and are further differentiated until stage 6, defined by the acquisition of CD57, thus being considered terminally differentiated NK cells [102,103]. Through this final differentiation step, CD56^dim^ NK cells loss NKG2A expression and sequentially acquire KIRs and CD57. CD57 expression increases with the expression of KIRs and correlate inversely with the expression of NKG2A. Moreover, they experience a gradual decline in proliferative capacity, along with different expression patterns of homing molecules [104,105]. Terminally differentiated NK cells are less responsive to stimulation by cytokines, but produce IFNγ and have a potent lytic activity when stimulated via CD16, evidencing a gradual shift in functionality during differentiation [103].

Although these NK cell developmental steps are well accepted, NK cell development is a more intricate process. For instance, different studies have revealed the existence of multiple cellular intermediates, such as unconventional CD56^dim^CD16^−^ NK cells [31] and CD56^bright^CD16^+^ NK cells [106]. Moreover, although in lower frequencies, it is possible to find all combinations of NKG2A and KIR expression in either CD57+ or CD57− CD56^dim^ NK cells [104]. Thus, it is obvious that the linear model of NK cell development is only an oversimplification of what really happens. Finally, little is known about the developmental origin of other NK cell subsets, such as adaptive NK cells [107] or CD56^neg^ NK cells, opening the possibility of yet not explored differentiation pathways.

## 5. The Role of NK Cells in Autologous Hematopoietic Stem Cell Transplantation

### 5.1. Hematopoietic Stem Cell Transplantation: Types and Indications

HSCT is a worldwide-established treatment option for many hematological disorders. In this type of cell immunotherapy, after the administration of an appropriate conditioning regimen that will ablate the recipient’s own bone marrow and induce sufficient immunosuppression to allow engraftment, HSCs are infused to replace the recipient’s unhealthy native bone marrow cells and immune system.

HSCT can be classified in different types based on the relationship between donor and recipient and the source of the graft. Thus, HSCT can be classified as autologous HSCT (autoHSCT), when the patient’s own stem cells are collected and reinfused at a later time, or alloHSCT, when HSCs come from another individual. In this latter scenario, donors can be related or unrelated HLA-matched. Moreover, when such donor cannot be found, umbilical cord blood (CB) or a related half-matched donor, also known as haploidentical transplantation, in which only one of the two HLA haplotypes is matched, can be used. On the other hand, according to the source, HSCs can be obtained from bone marrow, peripheral blood, or CB. Although HSCs were first harvested from bone marrow, nowadays and due to the development of mobilization procedures, such as the use of granulocyte colony-stimulating factor (G-CSF), peripheral blood is the main source of precursors [108,109].

In 2012, a total of 68,146 HSCTs were reported worldwide. Among them, 47% were allogenic and 53% autologous [110]. Moreover, in 2018, 47,468 transplants were reported only in Europe. Of these, 41% were allogenic and 59% autologous. In Europe, the main indications for alloHSCT are myeloid malignancies, being AML the largest indication, while, the main indications for autoHSCT are lymphoid malignancies, being multiple myeloma (MM) and non-Hodgkin lymphoma (NHL) the largest indications [111].

### 5.2. Factors Affecting autoHSCT Outcome

The benefits of alloHSCT go beyond the recovery from bone marrow aplasia after conditioning regimen, as immune cells in the graft might recognize and eliminate residual malignant cells, which is known as graft-versus-tumor (GvT) effect. Conversely, for years, autoHSCT has only been seen as a form for bone marrow rescue, necessary for hematologic engraftment, and its antitumor effect has been thought to rely on the conditioning regimen. Nevertheless, studies carried out in the last 15–20 years pointed out that a GvT effect might also be possible in autoHSCT setting [112]. As in alloHSCT, early immune recovery after autoHSCT has been associated with prolonged survival in a wide range of hematological malignancies [113,114,115,116]. Particularly, day 15 absolute lymphocyte count (ALC-15) of ≥500 cells/µL after autoHSCT was reported as an independent prognostic indicator for overall survival (OS) and progression-free survival (PFS) in MM and NHL patients [114]. Moreover, while different studies have not found any correlation between CD34+ cells dose and absolute lymphocyte count (ALC) recovery [117,118,119], a strong correlation was identified between the number of passively infused lymphocytes in the peripheral blood autograft (A)-ALC and ALC-15, being A-ALC higher in patients achieving an ALC-15 of ≥500 cells/µL [120]. The amount of passively transferred lymphocytes correlated inversely with the time to achieve a lymphocyte count of 0.5 × 10^9^ cells/L [118]. Furthermore, A-ALC was reported to be an independent prognostic factor for OS and PFS, in NHL patients [120]. T cells and NK cells were the main lymphocyte subsets identified in the autograft, and among them, a strong correlation was found between NK cells absolute numbers from the apheresis product and ALC-15 [117]. After that, NK cells were identified as the key lymphocyte subset in ALC-15 that impacted the outcome after autoHSCT. Thus, NHL patients with NK cell count at day 15 (NK-15) of ≥80 cells/µL experienced superior 3-year OS and PFS [121] and MM patients with NK cell count at 1 month of ≥100 cells/µL experienced prolonged PFS [122] than patients with lower counts. In addition, NHL patients with IL-15 levels ≥ 76.5 pg/mL at day 15 post-autoHSCT experienced superior 3-year OS and PFS compared with those patients with lower IL-15 levels. However, this survival benefit was reported to be most likely mediated by enhanced NK cell recovery after transplant [123]. Although all these are circumstantial evidences, and thus do not prove causality, they all suggest that the infusion of autograft lymphocytes does impact not only on immune reconstitution but also on the clinical outcome after autoHSCT (Table 2). Residual disease progression may be controlled by an early immune reconstitution and passively infused lymphocytes, opening the possibility for an autologous GvT effect [124,125].

### 5.3. Immune Reconstitution after autoHSCT and the Relevance of NK Cells

After HSCT, clinical engraftment is commonly considered when achieving a peripheral blood neutrophil count of >0.5 × 10^9^/L [131]. However, this does not mean that complete immune reconstitution occurs at this point. Reconstitution of immunologically competent effector cells occurs very gradually and more than a year may be needed to restore normal humoral and cellular immunity. Of note, B cells are barely detectable during the first 2–3 months post-HSCT, and the recovery of normal B cell numbers and functions can take up to 18 months [125]. Moreover, T cell recovery is also delayed, being CD8+ T cells’ restoration faster than CD4+ T cells, as the latter may fully reconstitute after 1-year post-HSCT. Thus, an inverted CD4/CD8 ratio is reported after HSCT [125].

In contrast, NK cells have been reported to be the first lymphocyte subset to return to normal levels, being the counts and functionality of NK cells restored as early as day 14 post-HSCT [125,132]. Considering that NK cells can display anti-tumor function independently of prior sensitization, play a role in immune surveillance and recover early after HSCT, the study of the reconstitution of these cells in autoHSCT settings is of utmost interest. Moreover, methods to improve NK cell recovery and post-HSCT immunomodulation targeting NK activity may also be of great interest. As mentioned before, the GvT effect exerted by NK cells in alloHSCT mainly relies in a mismatch between KIRs expressed by donor NK cells and the HLA class I ligands expressed on the recipient’s tumor cells [133,134]. As genes that encode KIRs and their HLA class I ligands segregate independently, expression of inhibitory KIRs but not its corresponding ligands, and vice versa, may happen. Although NK cells expressing inhibitory KIRs for non-self HLA molecules are hyporesponsive in steady state, in certain situations like inflammation, they may become responsive and play biologically important roles [135,136]. Thus, even though far less reported and studied, inhibitory KIR-HLA receptor-ligand mismatch can also occur in autoHSCT and affects patient’s outcome (Table 2). *HLA-Cw8* was reported to be an independent risk factor for poor outcome in lymphoma patients undergoing autoHSCT [126]. Moreover, lower risk of relapse was found when inhibitory KIR-HLA receptor ligand mismatch occur in a cohort of patients undergoing autoHSCT for solid tumor and lymphoma [127]. A similar outcome was also seen in patients undergoing autoHSCT for high-risk neuroblastoma. Particularly, patients lacking HLA-C1, the ligand for KIR2DL2/KIR2DL3, have the highest 3-years survival rate [128]. However, Stern and colleagues were not able to show that missing KIR-ligand effect in their autoHSCT cohort [137]. This is probably due to the fact that, in contrast to Leung et al., they grouped patients exclusively based on the presence or absence of KIR ligands and did not take into account inhibitory KIRs expression. In a cohort of AML patients treated with autoHSCT, those with *KIR* and *HLA* genotypes predictive of low-affinity interactions (*KIR3DL1^+^* and *HLA-Bw4-80Thr^+^*, *HLA-Bw4-80Ile*^−^ genotype) had lower incidence of relapse than patients with genotypes predictive of high-affinity interactions (*KIR3DL1^+^* and *HLA-Bw4-80Ile^+^* genotype), and this effect was also influenced by *HLA-Bw4* copy number [129]. This can be explained because HLA-Bw4-80Thr molecules bind KIR3DL1 with lower affinity than HLA-Bw4-80Ile, endowing lower responsiveness but weaker NK cell inhibition [129,138]. Finally, MM patients who were *KIR3DS1+* (activating KIR encoding gene) experienced shorter PFS after autoHSCT and this was more evident in patients that were at complete or partial remission at transplantation and who lacked the ligand for the inhibitory *KIR3DL1*, i.e., *HLA-Bw4* [130]. Although the mechanism by which *KIR3DS1* affects patient’s survival is not well understood, one possible explanation for its negative impact could be that *KIR3DS1+* NK clones may exert an immunomodulatory effect of antitumor responses by the production of anti-inflammatory cytokines [130]. On the other hand, it has been suggested that *KIR3DS1* accomplish a protective role in other human diseases [139]. Thus, this KIR might have either detrimental or beneficial effect depending on the type of disease.

**Figure 2:**
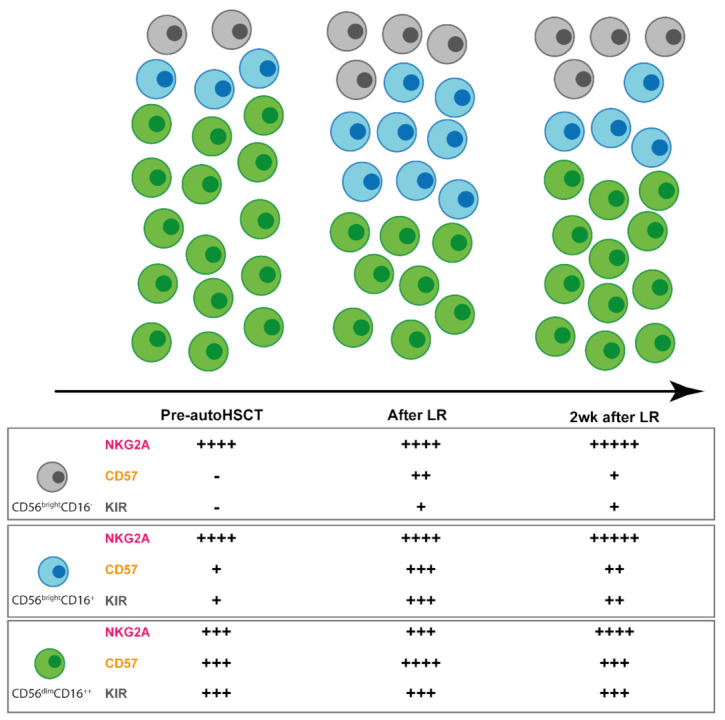
NK cell subset and receptor repertoire redistribution during NK cell reconstitution after autologous hematopoietic stem cell transplantation (autoHSCT). The distribution of the three NK cell subsets (CD56^bright^CD16^−^, CD56^bright^CD16^+^, and CD56^dim^CD16^++^) changes significantly at leucocyte recovery and some changes are still evident 2 weeks after leucocyte recovery. Moreover, the frequency of NK cells expressing the surface receptors NKG2A, CD57, and KIR in the different NK cell subsets is also altered. The frequency of NKG2A+, CD57+, and killer cell immunoglobulin-like receptors (KIR)+ NK cells within each subset is represented with the + symbol. After LR: after leucocyte recovery; 2 weeks after LR: 2 wk after leucocyte recovery.

As KIRs are just a minor part of the complex NK cell receptor repertoire, the study of further receptors, as well as the reconstitution of different NK cell subsets that might be implicated in the survival of patients undergoing autoHSCT is of great importance. In this sense, recently, whether a particular immune signature was associated with long-term complete response in MM patients was investigated. The authors found that, even if the frequency of NK cells within total lymphocytes was similar in patients and healthy donors, higher frequency of the inhibitory receptor KIR2DL1-expressing NK cells, higher frequency of NKG2A+ NK cells and lower frequency of NKp46+ NK cells were observed in MM patients in long-term complete response. Thus, a particular redistribution of NK cell activating and inhibitory receptors occurs in these patients [140]. In another recent study, Bhutani et al. examined the differences in terms of NK cell numbers and phenotype between MM patients with minimal residual disease (MRD) positive status versus MRD negative status after autoHSCT. These authors found that absolute numbers of NK cells were lower in patients with MRD positive response. In addition, and although the differences were not statistically significant, these patients have a higher frequency of KIRDS4+ NK cells and a lower frequency of NKG2A+ NK cells compared to patients with MRD-negative response [141]. Moreover, a few years ago, a comprehensive study of different NK cell subsets, their phenotype, and their function at three different time points, i.e., before autoHSCT (T1), after leukocyte regeneration (T2), and after 2 weeks of leukocyte recovery (T3), in patients undergoing autoHSCT was carried out (Figure 2) [142]. In this study, authors found that the frequency of NK cells within the leukocyte population significantly decreases from T1 to T2 but then recovered to the pre-autoHSCT levels at T3. Furthermore, the fold change ratio T2/T1 of the NK cell percentage was different between patients having a period time of ≤11 days between autoHSCT and T2 and those having a period of >11 days and also in patients with recurrent or refractory disease and those without. Focusing on NK cell subsets, they observed that CD56^dim^ (CD56^+^CD16^++^) subset was the main population at T1 and T3. However, NK cell subsets redistribution was observed at T2, in which the frequency of CD56^dim^ decreased, while the two populations of CD56^bright^ that they distinguish (CD56^++^CD16^−^ and CD56^++^CD16^+^) significantly increased, being the percentage of CD56^bright^CD16^+^ subset similar to the one of CD56^dim^ subset. The NK cell subset distribution at T3, although similar to the one observed at T1, was not the same. In addition, they studied the expression of NKG2A, CD57, and KIRs, i.e., markers related to NK cell differentiation and education. They found that on total NK cells, the frequency of NKG2A+ NK cell increased from T1 to T2 and was maintained elevated at T3. Furthermore, the expression of CD57 also increased from T1 to T2 but then recover to the initial levels at T3. A similar pattern of expression was also observed when the three subsets were separately analyzed. Particularly, the expression of CD57 at T2 in both CD56^bright^ subsets was surprising, as CD57 is considered a marker of terminally differentiation and CD56^bright^ are thought to be immature NK cells. In contrast, the frequency of KIR-expressing NK cells remains constant during NK cell reconstitution, even though the percentage of KIR+ cells in both CD56^bright^ subsets increased at T2 and maintained elevated at T3 (Figure 2). The elevated frequency of both CD57 and KIRs in CD56^bright^ subsets was found to be age dependent. They also performed an exhaustive study of different KIRs revealing that KIR2DL2/3/S2 and KIR3DL1 were upregulated in both CD56^bright^ subsets from T1 to T2 and that while KIR2DL2/3/S2 was the dominant KIR subset expressed at T1 on CD56^bright^CD16^−^ NK cells, KIR3DL1 was the dominant one at T2. In contrast, KIR2DL1/S1 levels remained constant. This pattern of KIR expression, in which KIR2DL2/3 and KIR3DL1 are the first KIRs appearing after HSCT, while KIR2DL1 is upregulated later, have been also observed in alloHSCT [143,144]. Finally, when effector functions of NK cells were assessed, Jacobs et al. reported that NK cells were able to degranulate (CD107a) and to produce cytokines (IFNγ) and chemokines (MIP1β) upon target cell recognition early after autoHSCT [142]. As the cohort of patients analyzed in this study is quite heterogeneous in terms of the type of cancer, it must be elucidated whether the NK cell subsets distribution, their phenotype and functionality during NK cell reconstitution will be the same in a particular hematological malignancy. In this sense, Jacobs et al. also found some differences regarding the CD56^dim^/CD56^bright^ ratio and the frequency of CD56^bright^NKG2A^+^ cells at T2 between MM and lymphoma patients undergoing autoHSCT [142]. Analysis of different NK cell subsets distribution was also carried out in a cohort of MM undergoing autoHSCT. In this case, the authors found that a population of CD56^dim^CD16^low^ was expanded after 2 weeks post-transplant and returned to initial levels after a month post-HSCT. They also described that this subset displayed the highest capacity to degranulate in vitro against K562 and MM cell lines [145]. Thus, it would be highly valuable to elucidate whether this subset plays a fundamental role in the GvT effect and in the outcome of MM patients after autoHSCT. Furthermore, an exhaustive analysis of other NK cell subsets that have been associated with survival in patients undergoing alloHSCT is also needed in an autoHSCT setting. This is, e.g., the case of adaptive NK cells and CD56^neg^ NK cells. In studies performed with alloHSCT recipients, it has been suggested that CMV-expanded NKG2C+ NK cell population may exert anti-leukemic effect. There have been also described that early human CMV reactivation after allogenic HSCT was associated with reduced risk for relapse [146,147,148,149,150,151]. Moreover, adaptive NK cell expansion was associated with lower relapse in different HSCT settings [152,153]. In addition, an expansion of CD56^neg^ NK cells after different HSCT settings have been described [154,155,156]. In T cell-depleted haploidentical HSCT, CD56^neg^ appear to be functional and capable of GvT effect [156].

Since NK cells seem to play a fundamental role early after autoHSCT, different therapeutic approaches have been used trying to enhance NK cell functional activity early after autoHSCT. Among others, the administration of IL-2 after autoHSCT has been studied. In general, significant increase in NK cell counts were observed. Moreover, in vitro enhanced NK cell cytotoxic activity was observed when compared to controls [157,158]. However, even though IL-2-based immunotherapy can be safely administrated after autoHSCT, it failed to demonstrate improvement in the disease outcome [157,159,160]. In addition, infusion of haploidentical or CB-derived NK cells in the setting of autoHSCT has been also studied [161,162,163,164,165]. Although the infusion of these NK cells seems to be well-tolerated, future studies regarding survival benefits of these treatment strategies are needed. Thus, in-depth studies of currently available therapies and of additional strategies targeting NK cells and enhancing their anti-tumor effector functions would be worth to explore [166].

## 6. Concluding Remarks

NK cells reconstitution, as opposed to what happens to other lymphocytes, occurs early after autoHSCT. Moreover, as several studies have shown, not only the NK cell count after autoHSCT have an impact on patient’s outcome but also the reconstitution of different NK cells subsets and their phenotype may be of great importance. For this reason, studies analyzing other NK cells subsets rather than the conventional CD56^bright^ and CD56^dim^ subpopulations, in addition to other aspects of NK cells phenotype will contribute to the better understanding of NK cell reconstitution after autoHSCT. These studies may also be helpful to identify new biomarkers that may predict HSCT outcomes, allowing individualizing and further improving this therapy. Furthermore, although the GvT effect mediated by NK cells has been well-defined in alloHSCT, in-depth studies are also needed to clarify this point in autoHSCT. Thus, there are still unsolved questions regarding the potential beneficial role that NK cells may have in autoHSCT settings that are worthy to explore.

## Figures and Tables

**Table 1:** Inhibitory and activating killer cell immunoglobulin-like receptors (KIRs) and their human leukocyte antigen (HLA) class I ligands.

Inhibitory KIRs	Ligands
KIR2DL1	HLA-C C2
KIR2DL2	HLA-C C1
	HLA-B*46:01
	HLA-B*73:01
KIR2DL3	HLA-C C1
	HLA-B*46:01
	HLA-B*73:01
KIR2DL4	HLA-G
KIR2DL5	Unknown
KIR3DL1	HLA-B Bw4
	HLA-A*23
	HLA-A*24
	HLA-A*32
KIR3DL2	HLA-A*03
	HLA-A*11
	HLA-F
KIR3DL3	Unknown
**Activating KIRs**	**Ligands**
KIR2DS1	HLA-C C2
KIR2DS2	HLA-C C1
	HLA-A*11:01
KIR2DS3	Unknown
KIR2DS4	HLA-C*02:02
	HLA-C*04:01
	HLA-C*05:01
	HLA-C*01:02
	HLA-C*14:02
	HLA-C*16:01
	HLA-A*11:01
	HLA-F
KIR2DS5	HLA-C C2
KIR3DS1	HLA-F
	HLA-B*51

**Table 2:** Factors affecting autologous hematopoietic stem cell transplantation (autoHSCT) outcome.

Factors	Prognostic Indicator for	Pathology	References
Absolute lymphocyte count at day 15 (ALC-15)	OS, PFS	Breast cancer, MM, NHL, HL, AML.	[113,114,115,116]
Autograft absolute lymphocyte count (A-ALC)	OS, PFS	NHL	[120]
NK cell count at day 15 (NK-15)	OS, PFS	NHL	[121]
NK cell count at 1 month	PFS	MM	[122]
IL-15 levels at day 15	OS, PFS	NHL	[123]
*HLA-Cw8* genotype	OS	Lymphoma	[126]
KIR-HLA receptor-ligand mismatch	Disease progression	Lymphoma, solid tumor, neuroblastoma	[127,128]
KIR and HLA genotypes predictive of low-affinity interactions	Relapse	AML	[129]
*KIR3DS1* genotype	PFS	MM in complete response or partial remission at autoHSCT	[130]

AML: acute myeloid leukemia; HL: Hodgkin lymphoma; IL-15: interleukin 15; MM: multiple myeloma; NHL: non-Hodgkin lymphoma; OS: overall survival; PFS: progression free survival.

## Data Availability

The data presented in this study are available on request from the corresponding author.
